# The experiences of homecare workers in navigating the COVID-19 pandemic: a protocol for a qualitative study

**DOI:** 10.12688/hrbopenres.13561.1

**Published:** 2022-06-07

**Authors:** Eva Moynan, Caitriona Lavelle, Katie Robinson, Pauline Boland, Pauline Meskell, Rose Galvin, Christine Fitzgerald

**Affiliations:** 1 School of Allied Health, Faculty of Education and Health Sciences, University of Limerick, Limerick, Ireland; 2 Clarecare, Ennis, Co Clare, V95F8CN, Ireland; 3 Ageing Research Centre, Health Research Institute, University of Limerick, Limerick, Ireland; 4 Department of Nursing and Midwifery, Faculty of Education and Health Sciences, University of Limerick, Limerick, Ireland

**Keywords:** Homecare workers, COVID–19 pandemic, experiences, qualitative.

## Abstract

**Background:** Globally, there have been over 400 million confirmed cases of coronavirus disease 2019 (COVID–19), including over 6 million deaths, reported to the World Health Organization. Older adults have been disproportionally affected by COVID-19 in terms of morbidity and mortality. Homecare workers continue to play a key role in supporting vulnerable people to live in their own homes. Unlike other health professionals, whose interactions with patients are relatively brief, homecare workers sometimes spend hours with clients assisting with caregiving and functional tasks. In addition, these workers frequently provide companionship and emotional support. The COVID-19 pandemic has resulted in many challenges to this caregiving role given the risk of virus transmission to both clients and homecare workers in the community. Despite the vital role homecare workers have played, qualitative research exploring perspectives of homecare workers experiences’ of providing help and care to older adults during the pandemic is sparse. This study aims to explore the experiences of homecare workers in navigating the COVID-19 pandemic.

**Methods:** A qualitative interpretative approach will be applied in this study through the facilitation of focus groups with homecare workers. The Consolidated Criteria for Reporting Qualitative Studies (COREQ) guidelines will be used to standardise the conduct and reporting of the research. Homecare workers will be recruited from a provider of homecare services in the Mid-West of Ireland (Clarecare) by a gatekeeper. Focus groups will be transcribed and analysed using a reflexive thematic approach supported by the use of NVIVO software package (Version 12).

**Conclusion: **This study represents a necessary first step in the development of an evidence base for clinical, education, and support needs of homecare workers.

## Introduction

Increased life expectancy, multimorbidity and population growth have led to an increase in the number of people who require homecare assistance to meet their health needs ( Martínez-Linares *et al.*, 2020). The homecare sector is projected to grow significantly as society ages and care shifts from the acute care to the home environment ( Leverton *et al.*, 2021). As the demand on the homecare sector increases, the sector is increasingly under pressure to retain experienced staff, and frequent turnover can negatively impact on the quality, safety and continuity of care, particularly among vulnerable older adults ( Burton *et al.*, 2022; Yeh *et al.*, 2019). The reasons for high turnover are multifactorial. Homecare workers are generally low paid, low status, have high staff turnover and are reliant on temporary or migrant staff, where training is not rewarded, mandatory or culturally valued ( D’Astous *et al.*, 2019; Vandrevala *et al.*, 2016; Zeytinoglu & Denton, 2006). It has been known to be called a “diaphanous profession” or the “invisible welfare” due to the lack of recognition these workers receive for their job ( Gazzaroli *et al.*, 2020). In a recent study, Belgian homecare workers described feeling “squeezed like a lemon” due to the poor working conditions and the demanding nature of the job ( Bensliman *et al.*, 2021). In Norway, homecare workers reported being exposed to occupational hazards and physically demanding tasks which affected their safety, health and wellbeing ( Gronoset Grasmo *et al.*, 2021). In the UK, qualitative semi-structured interviews were held with 29 homecare workers providing care to people with dementia at end of life and 13 homecare managers. The homecare workers felt “emotionally drained, under-prepared and overwhelmed”. It was found that there is a need for development of models to support homecare workers and alleviate the emotional, interpersonal and practical burdens ( Yeh *et al.*, 2019, pp 352–359). A further UK study explored the training and support needs of homecare workers and reinforced the need for the development of a professional framework, incorporating training for homecare workers as well as ongoing practical and emotional support ( Leverton *et al.*, 2021).

In Ireland, a home support service is provided to older adults who wish to remain in their own home. This service was previously known as the Home Help Service or Home Care Package. The individuals caring for older adults are known as homecare workers, home carers, home help or home support ( Home Support Services, 2022). In 2018, the Institute of Public Health in Ireland conducted a report to improve home care services in Ireland ( The Institute of Public Health, 2018). This report highlighted that home carer workers are largely underpaid; employment is often insecure and opportunities for advancement are low. It was recommended that homecare be defined, and specific services should be addressed i.e., sociopsychological needs, loneliness, companionship and domestic services. In addition, it was recommended that a standardised policy framework may be important to implement, however, there must be scope for service flexibility.

Homecare workers have played a key role in supporting community dwelling older adults to live in their own homes and communities during the coronavirus disease 2019 (COVID-19) pandemic. The pandemic has led to 400 million reported cases and 6 million deaths globally ( WHO, 2022). Unlike other health professionals, whose interactions with patients are relatively brief, homecare workers sometimes spend hours with clients assisting with caregiving and functional tasks. In addition, these workers frequently provide companionship and emotional support. The COVID-19 pandemic has resulted in many challenges to this caregiving role, given the risk of virus transmission to both clients and homecare workers in the community. A US study found the COVID-19 pandemic led to deterioration of clients’ health due to a lack of social supports compared to those available pre pandemic, with this paucity of support networks found to impact mental and physical health, particularly in the role of homecare workers ( Bell *et al.*, 2022). These findings echoed a study exploring COVID-19 challenges amongst home based palliative care workers, with staff driven to provide care throughout COVID-19 and its associated risks due to a strong sense of responsibility towards their clients and colleagues, identifying their role as one of the few stable supports for clients and families ( Franchini *et al.*, 2021). Insights for an EU study which explored homecare workers’ training and learning experiences during COVID-19 highlighted the challenges faced by staff, while also acknowledging homecare workers’ adaptability through the learning of new practices, skills and approaches ( Malmgren Fänge *et al.*, 2022).

Despite the recognition of higher mortality rates among older adults and higher overall rates of disease among nursing home staff, little remains known about the risks and experiences of workers who provide help and care to older adults who live at home ( Allison *et al.*, 2020; Weeks *et al.*, 2021). The protocol for this qualitative study explores the experiences of homecare workers in navigating the COVID-19 pandemic.

## Protocol

### Design

A qualitative interpretative design will be adopted to explore in-depth the experiences of homecare workers during the COVID-19 pandemic. This study design was chosen as it is effective for intervention development in the way it allows the focus of the study to be on the homecare workers’ experiences. This approach is useful when collecting homecare workers’ views on this specific topic. This study will adhere to the Consolidated Criteria for Reporting Qualitative Research (COREQ) checklist for interviews and focus groups to ensure rigour and comprehensiveness ( Tong *et al.*, 2007).

### Ethics

Ethical approval was granted from the Research Ethics Committee of the Faculty of Education and Health Sciences, University of Limerick (Ref: 2021_12_18_EHS). Date of grant approval 22/10/21. Written consent forms, along with study information sheets, will be distributed among participants. The study information sheets have been approved by the ethics committee. The protocol on Dealing with Distressed participants will be adhered to should sensitive topics be brought up ( Moynan *et al*., 2022b).

### Recruitment and participants

Homecare workers will be purposively recruited from Clarecare. Clarecare operates a homecare service in the Mid-West of Ireland (mainly in County Clare) on behalf of the national Health Service Executive (HSE). This service mainly provides homecare assistance to older people. The organisation currently employs 279 homecare workers, providing care to adults aged ≥65 years in the MidWest.

Homecare staff will be recruited through an email invitation from a gatekeeper in Clarecare. Inclusion and exclusion criteria will be developed to support methodological rigour. Inclusion criteria: A) Adults aged ≥18 years; B) Homecare worker has the capacity and willingness to provide informed consent; C) Homecare worker working with older adults ≥65 years. Exclusion criteria: A) Homecare worker exclusively working with younger populations (<65 years); B) Inability to communicate sufficiently in English to participate in focus groups.

When prospective participants express interest in participating, the research team will arrange a follow up phone call a minimum of two days later to allow time to consider participating. It is anticipated that three focus groups will be facilitated, with 6–8 homecare workers participating in each focus group, providing insights from homecare workers across five geographic areas. 6 – 8 homecare workers in each focus group is in line with supporting literature on the facilitation of focus groups which reflects the importance of a smaller group to facilitate effective engagement and allow opportunities for participation amongst participants ( Braun & Clarke, 2021; Kreuger & Casey, 2000).

## Data collection

Study researchers developed a semi-structured focus group guide which consists of three content areas: participants’ experiences of providing homecare since the COVID-19 outbreak, challenges participants have faced since the COVID-19 outbreak, what has supported participants since the COVID-19 outbreak. The focus group guide consists of open-ended questions and related prompts. The focus groups will take place in person in mid-June in 3 Clarecare bases – Killaloe, Kilkee and Ennis, over a period of 3 days. See extended data for the focus group guide ( Moynan *et al*., 2022a).

## Data analysis

Focus groups will be digitally audio-recorded, transcribed and exported to NVivo (Version 12.6.0). Each participant will get their own unique number. The participants’ details and their associated numbers will be kept on a password-protected Excel file. The transcribed focus group data will be analysed following a reflexive thematic approach ( Braun & Clarke, 2021) through the identification and analysis of themes from the focus group data ( Byrne, 2021). Initially a focus group transcript will be coded by two independent researchers (EM and CF) and subsequently discussed to explore different perspectives and insights from coders, as opposed to seeking consensus. All data will then be coded by one researcher. The six steps of thematic analysis described by Braun and Clarke will be followed ( Braun & Clarke, 2021; Braun & Clarke, 2006), which will be used as a guide, with an emphasis on the flexible nature of these steps in line with the reflective thematic analysis approach utilised. The first step, involving data familiarisation, will be achieved through analysis following transcription and reading of all data. The second step, involving generating initial codes systematically across the data set, will have a particular focus on the research question. In the third step, overlapping codes will be identified and preliminary themes will be identified. In the fourth step, these themes will be reviewed with the research supervisor and in the fifth step they will be defined and named. The final step involving preparation of the manuscript with quotes from the focus group data will be completed to illustrate the themes identified.

## Dissemination

Subsequent to the analysis stage, key themes and a summary of findings will be shared with participants. Abstracts will be submitted to relevant national and international conferences. Locally, the study results will be shared with management and staff at Clarecare.

## Study status

Data collection is expected to take place mid-June 2022, with transcription of interviews and data analysis to be completed late June 2022.

## Conclusion

To the authors’ knowledge, no studies have been conducted to explore the experiences and perspectives of homecare workers during the COVID-19 pandemic. It is anticipated that understanding and exploring their experiences will assist in the development of interventions that may serve to improve working conditions and supports for homecare workers.

## Data availability

### Underlying data

No underlying data are associate with this article.

### Extended data

figshare: Appendix 1.docx. https://doi.org/10.6084/m9.figshare.19830145.v1 ( Moynan *et al.*, 2022a).

This project contains the focus group guide.

figshare: Dealing with Distressed Participants. https://doi.org/10.6084/m9.figshare.19878754.v1 ( Moynan *et al.*, 2022b).

This project contains the ‘Dealing with Distressed Participants’ guide and further resources.

Data are available under the terms of the Creative Commons Attribution 4.0 International license (CC-BY 4.0).

## References

[ref-1] Allison TA Oh A Harrison KL : Extreme Vulnerability of Home Care Workers During the COVID-19 Pandemic—A Call to Action. *JAMA Intern Med.* 2020;180(11):1459–1460. 10.1001/jamainternmed.2020.3937 32749452 PMC7858686

[ref-3] Bell SA Krienke L Brown A : Barriers and facilitators to providing home-based care in a pandemic: policy and practice implications. *BMC Geriatr.* 2022;22(1):234. 10.1186/s12877-022-02907-w 35313830 PMC8936035

[ref-4] Bensliman R Casini A Mahieu C : “Squeezed like a lemon”: A participatory approach on the effects of innovation on the well-being of homecare workers in Belgium. *Health Soc Care Community.* 2021. 10.1111/hsc.13506 34250683

[ref-5] Braun V Clarke C : Using Thematic Analysis in Psychology. *Qual Res Psychol.* 2006;3(2):77–101. 10.1191/1478088706qp063oa

[ref-6] Braun V Clarke V : Can I use TA? Should I use TA? Should I not use TA? Comparing reflexive thematic analysis and other pattern-based qualitative analytic approaches. *Couns Psychother Res.* 2021;21(1):37–47. 10.1002/capr.12360

[ref-7] Burton E Horgan NF Cummins V : A Qualitative Study of Older Adults' Experiences of Embedding Physical Activity Within Their Home Care Services in Ireland. *J Multidiscip Healthc.* 2022;15:1163-1173. 10.2147/JMDH.S351714 35615293 PMC9126230

[ref-8] Byrne D : A worked example of Braun and Clarke's approach to reflexive thematic analysis. *Quality & Quantity.* 2021;1–22. 10.1191/1478088706qp063oa 33879929

[ref-11] D’Astous V Abrams R Vandrevala T : Gaps in Understanding the Experiences of Homecare Workers Providing Care for People with Dementia up to the End of life: A Systematic Review. *Dementia (London).* 2019;18(3):970–989. 10.1177/1471301217699354 28358269

[ref-13] Franchini L Varani S Ostan R : Home palliative care professionals perception of challenges during the Covid-19 outbreak: A qualitative study. *Palliat Med.* 2021;35(5):862–874. 10.1177/02692163211008732 33829909

[ref-14] Gazzaroli D D’Angelo C Corvino C : Home-care Workers’ Representations of Their Role and Competences: A Diaphanous Profession. *Front Psychol.* 2020;11:581399. 10.3389/fpsyg.2020.581399 33362645 PMC7758209

[ref-15] Gronoset Grasmo S Ingeborg Liaset F Redzovic SE : Home care workers' experiences of work conditions related to their occupational health: a qualitative study. *BMC Health Serv Res.* 2021;21(1):962. 10.1186/s12913-021-06941-z 34521407 PMC8438557

[ref-16] Home Support Services.HSE,2022. Reference Source

[ref-17] Kreuger RA Casey MA : Focus Groups: A Practical Guide for Applied Research, 3 ^rd^.ed., CA: Sage Publications.2000. Reference Source

[ref-18] Leverton M Burton A Beresford-Dent J : ‘You can’t just put somebody in a situation with no armour’. An ethnographic exploration of the training and support needs of homecare workers caring for people living with dementia. *Dementia (London).* 2021;20(8);2982–3005. 10.1177/14713012211023676 34111969 PMC8678657

[ref-19] Malmgren Fänge A Christensen J Backhouse T : Care home and home care staff’s learning during the COVID-19 pandemic and beliefs about subsequent changes in the future: A survey study in Sweden, Italy, Germany and the United Kingdom.In *Healthcare (Basel).* MDPI,2022;10(2):306. 10.3390/healthcare10020306 35206920 PMC8872186

[ref-20] Martínez-Linares JM Andújar-Afán FA Martínez-Yébenes R : A Qualitative View of Homecare Support Workers on Unmet Health Needs of People with Dependency. *Int J Environ Res Public Health.* 2020;17(9);3166. 10.3390/ijerph17093166 32370123 PMC7246884

[ref-21] Moynan E Galvin R Robinson K : Appendix 1.docx. figshare.Journal contribution. [Data].2022a. 10.6084/m9.figshare.19830145.v1

[ref-22] Moynan E FITZGERALD C Galvin R : Dealing with Distressed Participants. figshare. Journal contribution. [Data].2022b. 10.6084/m9.figshare.19878754.v1

[ref-23] The Institute of Public Health in Ireland: Improving Home Care Services in Ireland: An Overview of the Findings of the Department of Health's Public Consultation.Dublin: Institute of Public Health in Ireland.2018. Reference Source

[ref-24] Tong A Sainsbury P Craig J : Consolidated criteria for reporting qualitative research (COREQ): a 32-item checklist for interviews and focus groups. *Int J Qual Health Care.* 2007;19(6):349–357. 10.1093/intqhc/mzm042 17872937

[ref-25] Vandrevala T Samsi K Rose C : Perceived needs for support among care home staff providing end of life care for people with dementia: a qualitative study. *Int J Geriatr Psychiatry.* 2016;32(2);155–163. 10.1002/gps.4451 26988707

[ref-26] Weeks LE Nesto S Hiebert B : Health service experiences and preferences of frail home care clients and their family and friend caregivers during the COVID-19 pandemic. *BMC Res Notes.* 2021;14(1):271. 10.1186/s13104-021-05686-6 34261523 PMC8278184

[ref-27] WHO: WHO Coronavirus (COVID-19) Dashboard.unpublished.2022. Reference Source

[ref-28] Yeh IL Samsi K Vandrevala T : Constituents of effective support for homecare workers providing care to people with dementia at end of life. *Int J Geriatr Psychiatry.* 2019;34(2):352–359. 10.1002/gps.5027 30430628

[ref-29] Zeytinoglu IU Denton M : Satisfied Workers, Retained Workers: Effects of Work and Work Environment on Homecare Workers’ Job Satisfaction, Stress, Physical Health, and Retention.Canadian, *Health Services Research Foundation.* 2006. Reference Source

