# Supplementary material for: The experiences of homecare workers in navigating the COVID-19 pandemic: a protocol for a qualitative study
Source: HRB Open Res. 2022 Jun 7;5:43. [Version 1] doi: 10.12688/hrbopenres.13561.1 (PMC10444994; doi:10.12688/hrbopenres.13561.1)
Supplement: Peer review history

# Peer review: Reviewer response for version 1

**DOI:** 10.21956/hrbopenres.14807.r34750

**Published:** 2023-08-22

**Authors:** Julien Mercille

**Affiliations:** 1 School of Geography, University College Dublin, Dublin, Ireland

A good project that is very worthwhile. A few comments to improve it:

- The Introduction takes time to get to the precise aim of the paper; I would foreground the aim and go from there.
- There is some literature on home care workers'  and nursing home workers' experiences during Covid that has appeared recently that should be referenced to better situate the study, and also to compare your findings to those studies--your project's Discussion section could address that, for example:
  - Mercille J, Edwards J, O'Neil N (2022) Home care professionals’ views on working conditions during the COVID-19 pandemic: the case of Ireland, International Journal of Care and Caring 6, 1-2: 85-102 (email me if you want a copy).
  - O'Neill, N, Edwards, J, Mercille, J (2023) Home care workers' views of employment conditions: private for-profit vs public and non-profit providers in Ireland, International Journal of Sociology and Social Policy, 43 (13-14): 19-35.
- Have you seen the NORDCARE survey that lists standard questions for home care workers? This could be used as it provides a way to compare and contrast your results to those of studies in other European countries.
- About the research design: Could you please elaborate on the gatekeeper? Is it the employer (Clarecare)? If so, my initial reaction would be that I would be careful about asking the employer to act as gatekeeper when questions and answers from the workers are likely to be critical of the employer. Is there another way to recruit workers?

Is the study design appropriate for the research question?

Yes

Is the rationale for, and objectives of, the study clearly described?

Yes

Are sufficient details of the methods provided to allow replication by others?

Yes

Are the datasets clearly presented in a useable and accessible format?

Not applicable

Reviewer Expertise:

Long-term care; political economy; social policy

I confirm that I have read this submission and believe that I have an appropriate level of expertise to confirm that it is of an acceptable scientific standard.

## References

[1] : Home care professionals’ views on working conditions during the COVID-19 pandemic: the case of Ireland. *International Journal of Care and Caring* .2022;6(1-2) : 10.1332/239788221X16345464319417 85-102 10.1332/239788221X16345464319417

[2] : Home care workers' views of employment conditions: private for-profit vs public and non-profit providers in Ireland. *International Journal of Sociology and Social Policy* .2023;43(13/14) : 10.1108/IJSSP-10-2022-0276 19-35 10.1108/IJSSP-10-2022-0276

# Peer review: Reviewer response for version 1

**DOI:** 10.21956/hrbopenres.14807.r32419

**Published:** 2022-08-11

**Authors:** Corina Naughton

**Affiliations:** 1 Catherine McAuley School of Nursing and Midwifery, University College Cork, Cork, Ireland

Thank you for the opportunity to review this interesting and important study protocol. The protocol is well written with a strong rationale and a good overview of international literature.

**Aim**

- The aim is very broad - this could be refined a bit more.

**Design and methods section**

- The design needs to be more clearly and accurately described. It seems the authors will use thematic analysis - this approach is largely descriptive. Interpretive descriptive (ID) is a distinct design (see Sally Thorne ^1^, Thompson Burdine *et al., *2021 ^2^). The authors need to be able to distinguish between these approaches with appropriate rationale. The thematic approach to coding is similar for both designs, but there is an additional level of interpretation based on the context in ID.
- In an ideal world, it is better to have time between FG to allow for preliminary coding and to identify new emerging topics that should be explored in subsequent FG interviews.
- Will the authors offer the opportunity for 1:1 or telephone interviews in the event a person would like to be involved but cannot attend or is afraid to speak openly given they are interviewed on their employer's premises - these are private, not public workers - it is worth considering how this risk can be managed.

**Abstract**

- A minor point: " *Unlike other health professionals*", home care workers don’t hold a professional registration – so query ‘health care workers‘.
- Define who you mean by home care workers - there are multiple terms used for this workforce, clarify it excludes community nurses, PHN, and volunteer visitors.
- " *A qualitative interpretative approach"* - This is not a qualitative design? Interpretive descriptive or thematic analysis – be more specific about design.

**Background**

- Define home care work (see comment in the abstract).
- " *Communities during the coronavirus disease 2019*" - 'Communities' does not seem to make sense in the context of this sentence.
- *"A US study found the COVID-19 pandemic led to deterioration of clients’ health due to a lack of social supports compared to those available pre pandemic, with this paucity of support networks found to impact mental and physical health, particularly in the role of homecare workers (Bell et al., 2022)". C*larify is it the client or home care workers whose physical and mental health deteriorated.
- " *little remains known*"- Rephrase.

**Design**

- Aim: the aim is buried in the design sentence, state the aim more clearly.
- The aim of the study is: - also useful to expand on objectives - for example, later you justify the design as an approach to intervention design, so is an objective to identify learning and emotional support needs?
- " *A qualitative interpretative design will be adopted to explore in-depth the experiences of homecare workers during the COVID-19 pandemic.*" - 'A qualitative interpretative design', this is not a design? Descriptive interpretive or descriptive phenomenon or thematic analysis – need to reference design.
- *"This study design was chosen as it is effective for intervention development in the way it allows the focus of the study to be on the homecare workers’ experiences*." - Need to reference. As a protocol, include more detail on the distinguishing features of the design you have chosen supported with references.
- " *homecare workers across five geographic areas" - *How are you defining a geographical region, the provider operates in one county - clarify.

**Inclusion criteria**

- Is there a minimum duration of working as a home care worker?
- Sample size: outline the role of data saturation in determining sample size.
- Outline how the topic guide was developed - the broad questions.
- Your design seems to be thematic analysis rather than interpretative descriptive – thematic analysis is both a design and approach to coding. Need for further clarification here.
- Clarify if interviews and analysis will be undertaken concurrently with the opportunity to modify or explore new concepts in later interviews or will the interviews all be completed prior to iterative analysis?
- Will there be any attempt to validate the themes with the homecare workers? Clarify if this is part of the analysis stage or just post-analysis dissemination.

To the authors’ knowledge, no studies have been conducted to explore the experiences and perspectives of homecare workers during the COVID-19 pandemic - is this in the Irish context? The background suggested there is international data.

Is the study design appropriate for the research question?

Yes

Is the rationale for, and objectives of, the study clearly described?

Yes

Are sufficient details of the methods provided to allow replication by others?

Partly

Are the datasets clearly presented in a useable and accessible format?

Not applicable

Reviewer Expertise:

Older adults

I confirm that I have read this submission and believe that I have an appropriate level of expertise to confirm that it is of an acceptable scientific standard.

## References

[1] : Interpretive Description: Qualitative Research for Applied Practice. *Routledge* .2016;

[2] : Interpretive description: A flexible qualitative methodology for medical education research. *Med Educ* .55(3) : 10.1111/medu.14380 336-343 10.1111/medu.14380 32967042
